# Clinical outcomes of obese and nonobese patients with atrial fibrillation according to associated metabolic abnormalities: A report from the COOL‐AF registry

**DOI:** 10.1111/1753-0407.13519

**Published:** 2023-12-14

**Authors:** Rungroj Krittayaphong, Thanita Boonyapiphat, Arjbordin Winijkul, Gregory Y. H. Lip

**Affiliations:** ^1^ Division of Cardiology, Department of Medicine, Faculty of Medicine Siriraj Hospital Mahidol University Bangkok Thailand; ^2^ Lampang Hospital Lampang Thailand; ^3^ Liverpool Centre for Cardiovascular Science University of Liverpool, Liverpool John Moores University and Liverpool Heart & Chest Hospital Liverpool UK; ^4^ Danish Center for Clinical Health Services Research, Department of Clinical Medicine Aalborg University Aalborg Denmark

**Keywords:** atrial fibrillation, metabolic healthy, obesity, outcomes

## Abstract

**Background:**

The primary objective was to determine the influence of obesity and associated metabolic status on clinical outcomes of Asian patients with atrial fibrillation (AF).

**Methods:**

This study was based on a prospective multicenter of patients with nonvalvular AF. Patients were classified as obese and nonobese and being metabolic unhealthy was defined as having at least one of the three cardiovascular risk factors including dyslipidemia, hypertension, or diabetes mellitus. Outcomes were a primary composite outcome of all‐cause death, ischemic stroke/systemic embolism (SSE), acute myocardial infarction (MI), and heart failure (HF), as well as the individual end points.

**Results:**

There were a total of 3141 enrolled patients (mean age 67.4 ± 11.1 years; 41.0% female), of whom 1566 (49.9%) were obese and 2564 (81.6%) were metabolic unhealthy. During a mean follow‐up of 32.2 ± 8.3 months, the incidence rate of the composite outcome, all‐cause death, SSE, MI, and HF were 7.21 (6.63–7.82), 3.86 (3.45–4.30), 1.48 (1.23–1.77), 0.47 (0.33–0.64), and 2.84 (2.48–3.23) per 100 person‐years, respectively. Metabolic unhealthy nonobese subjects were at higher risk of the composite outcomes than metabolic unhealthy obese subjects with hazard ratio (HR) 1.39, 95% confidence interval (CI) 1.17–1.66, *p* < .001. Metabolic unhealthy obese subjects tend to have an increased risk of the composite outcomes compared to those metabolic healthy obese (HR 1.36, 95% CI 0.91–2.02, *p* = .133). Metabolic healthy obese subjects were not associated with increased risk.

**Conclusions:**

Metabolic unhealthy obese subjects were associated with an increased risk of adverse outcomes in AF patients, whereas metabolically healthy obesity was not associated with an increased risk.

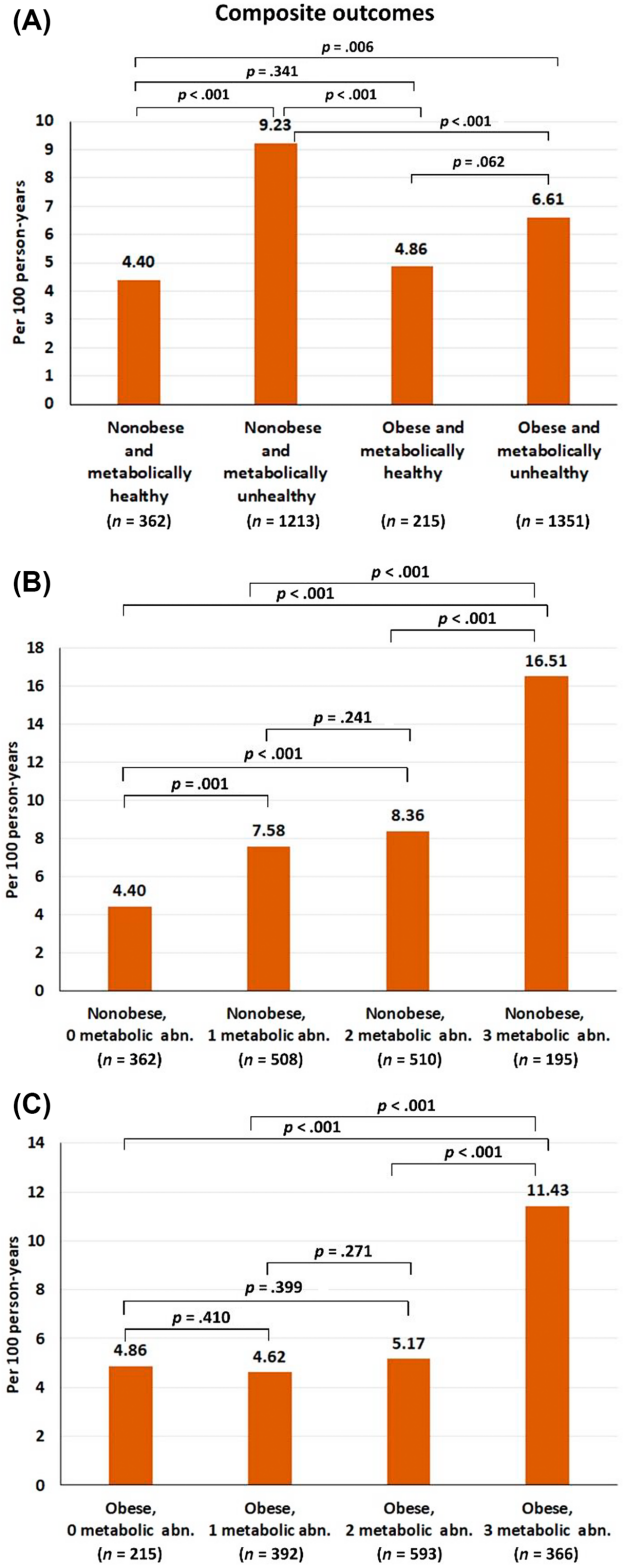

## Graphical abstract

**Highlights**

- The results of this prospective multicenter nationwide study showed that among patients with atrial fibrillation (AF), metabolic unhealthy obese subjects were associated with an increased risk of adverse outcomes in patients with AF.
- Obese patients with AF and good metabolic control or metabolically healthy obesity were not associated with an increased risk.

## 1 INTRODUCTION

In the general population, there appears to be an “obesity paradox” whereby obese patients seem to be associated with less adverse outcomes compared to nonobese patients.^1^, ^2^ However, obesity is not a binary (yes/no) risk, and unhealth metabolic features may influence outcomes. Indeed, in the general population both obesity and metabolic unhealthy features have been associated with poor prognosis. Obesity, especially abdominal obesity, is associated with an increased risk of cardiovascular events including coronary event, heart failure, stroke, and death.^3^ Cardiovascular risk factors are also more frequent in obese patients.^4^ In patients with atrial fibrillation (AF), an obesity paradox is also evident from various studies.^5^

With respect to obesity, the Asian population may be different from non‐Asians. For example, the Asian criterion for obesity was a body mass index (BMI) >25 kg/m^2^.^6^ Even in lean patients, Asians can have a greater proportion of visceral fat compared to non‐Asians.^7^, ^8^ Given these differences between Asian and non‐Asian subjects, the impact of obesity and/or metabolically unhealthy features on prognosis in patients with AF merits further study. This is important given the need for a more holistic or integrated care approach to better characterization^9^ and management of AF, which also includes symptom management and mitigation of cardiovascular risk factors and comorbidities, including lifestyle factors and obesity management.^10^, ^11^ Compliance with such an approach has been associated with improved clinical outcomes.^12^

The primary objective of this study was to determine the influence of obesity and metabolic status on clinical outcomes in patients with AF. The secondary objective was to determine the influence of metabolic status on clinical outcomes in obese and nonobese AF patients.

## 2 METHODS

### 2.1 Study population

This is a prospective registry of patients with nonvalvular AF. Inclusion criteria were patients aged >18 years with documented diagnosis of AF confirmed by 12‐lead ECG or ambulatory ECG monitoring. Exclusion criteria were as follows: (a) rheumatic mitral stenosis; (b) mechanical heart valves; (c) AF from transient cause; (d) prior ischemic stroke in 3 months; (e) hematologic conditions with increased risk of bleeding; (f) life expectancy less than 3 years; (g) participating in pharmaceutical clinical trial; (h) cannot attend for follow‐up visit(s); and (i) refusal to participate. Patients with missing BMI data or BMI in the underweight range defined as a BMI <18.5 kg/m^2^ were also excluded. The study was conducted in accordance with the principles in the Declaration of Helsinki and the International Conference on Harmonization for Good Clinical Practice Guidelines. The Central Research Ethics Committee approved this study. All patients gave written informed consent before the participation.

### 2.2 Study protocol

The site investigators were requested to enroll patients consecutively. The study population was selected from outpatient departments of internal medicine and cardiology clinics. The study team screened the inclusion and exclusion criteria. They approached the patients and explained the study details before asking patients to sign the informed consent. The study team collected baseline data after the consent process.

Site investigators reviewed medical records and conducted interviews with the study subjects to acquire the required information. Investigators recorded data in the recorded form and web system. Recorded forms were sent to the data management site by surface mail. The study team at the data management site or central site verified the data in the recorded form and on the web. The team at the central site sent queries to the study site investigators to verify or correct the data during the data verification process. The monitoring process was performed for every study site by the monitoring team to confirm data quality and ensure that the study investigators conducted the study in accordance with good clinical practice. Follow‐up data were recorded every 6 months up to 3 years.

### 2.3 Data collection

Researchers collected baseline demographic data; vital signs; body weight; height; BMI; AF types, duration, and symptoms; cardiovascular risk factors; history of comorbidities; data on laboratory study; ECG; echocardiogram; other investigations; and medications including antithrombotic medications. Data of metabolic conditions including hypertension, diabetes, and dyslipidemia were collected. Components of the scoring system were recorded as follows: CHA_2_DS_2_‐VASc score (C = congestive heart failure [1 point]; H = hypertension [1 point]; A = age ≥75 years [2 points]; D = diabetes mellitus [1 point]; S = stroke or TIA [2 points]; V = vascular disease [1 point]; A = age 65–74 years [1 point]; and Sc = female sex [1 point]); HAS‐BLED score (uncontrolled Hypertension, Abnormal renal, or liver function; history of Stroke; history of Bleeding; Labile INR; Elderly [age above 65 years]; and Drugs or alcohol [1 point each]). During the follow‐up visits, site investigators were required to record the presence and details of clinical outcomes. Other data were also collected in a similar manner as the baseline visit.

### 2.4 Outcomes and definitions

Diagnosis of hypertension was based on medical history and the use of antihypertensive medications. Hypertension was defined by systolic blood pressure ≥140 mm Hg and/or diastolic blood pressure ≥90 mmHg at least twice. The diagnosis of diabetes mellitus was based on the patient's history, medical records, and the use of glucose‐lowering medications. Diabetes mellitus was defined as a fasting plasma glucose level ≥126 mg/dL after a minimum of two tests or a glucose level of ≥200 mg/dL measured at any time during the day.

The primary outcome of this study was a composite of ‘all‐cause death, ischemic stroke/systemic embolism (SSE), acute myocardial infarction (MI), and heart failure’. Individual outcomes were also reported. Ischemic stroke or transient ischemic attack (TIA) were defined as acute focal neurological deficit lasting more than or less than 24 h for ischemic stroke or TIA, respectively. Imaging data from computed tomography brain scan or magnetic resonance imaging were required to be uploaded into the web‐based system but were not required to be positive for diagnosis. Systemic embolism needed both clinical and objective evidence of sudden loss of end‐organ perfusion for the diagnosis. Acute MI was defined as a detection of a rise and/or fall of cardiac troponin with at least one value above the 99th percentile upper reference limit and with at least one of the following: symptoms of ischemia, new significant ST–T changes or new left bundle‐branch block, development of pathological Q waves in the ECG, or imaging evidence of new loss of viable myocardium or new regional wall motion abnormality. Heart failure (HF) was defined as a hospital admission or a presentation of the patient for an urgent, unscheduled clinic/office/emergency department visit, with a primary diagnosis of HF, whereby the patient exhibits new or worsening HF symptoms, has objective evidence of HF, and received initiation or intensification of HF treatment.

The supporting documents used to confirm the primary outcome clinical event were uploaded into the web‐based system for event verification. All outcomes were adjudicated by a clinical events committee. Site investigators were requested to respond to queries for verification of clinical event. The adjudication committee assessed the supporting documents of each event and make consensus whether the outcome is confirmed.

### 2.5 Statistical analysis

Results are displayed as mean and SD for continuous data and count and percentages for categorical data. Comparisons of two groups were made by the Student *t* test for unpaired data for continuous data and the chi‐square test for categorical data. Obesity was defined as a BMI ≥25 kg/m^2^. Metabolic unhealthy was defined as having at least one abnormal metabolic factor (out of hypertension, diabetes, or dyslipidemia). Comparisons of four groups classified by the obesity and metabolic status were made by analysis of variance test for continuous data and the chi‐square test for categorical data. The incidence rate of clinical outcomes are shown as incidence rates per 100 person‐years. The comparisons of incidence rates were made by the Poisson test. Kaplan–Meier analysis with log‐rank test was performed to assess the survival free of clinical event. Age‐ and sex‐adjusted multivariable Cox proportional hazard model analysis was performed to assess the predictors for clinical outcomes using the time to event data. Sensitivity analysis was performed for the influence of BMI on composite outcome treating BMI as continuous data and shown as the restricted cubic spline graph. Cubic spline graphs was also used to test the interaction of BMI and metabolic status on composite outcome. A *p*‐value of <.05 indicated statistical significance. Statistical analysis was performed by the SPSS statistical software version 18.0 (SPSS, Inc., Chicago, IL, USA) and R version 3.6.3 (www.r-project.org).

## 3 RESULTS

This prospective nationwide AF registry was conducted in 27 hospitals in Thailand, with enrollment during 2014–2017. Among 3141 patients included in this analysis, mean age was 67.4 ± 11.1 years; 41.0% were female; 1566 were obese (49.9%), and 2564 were metabolic unhealthy (81.6%).

**FIGURE 1:**
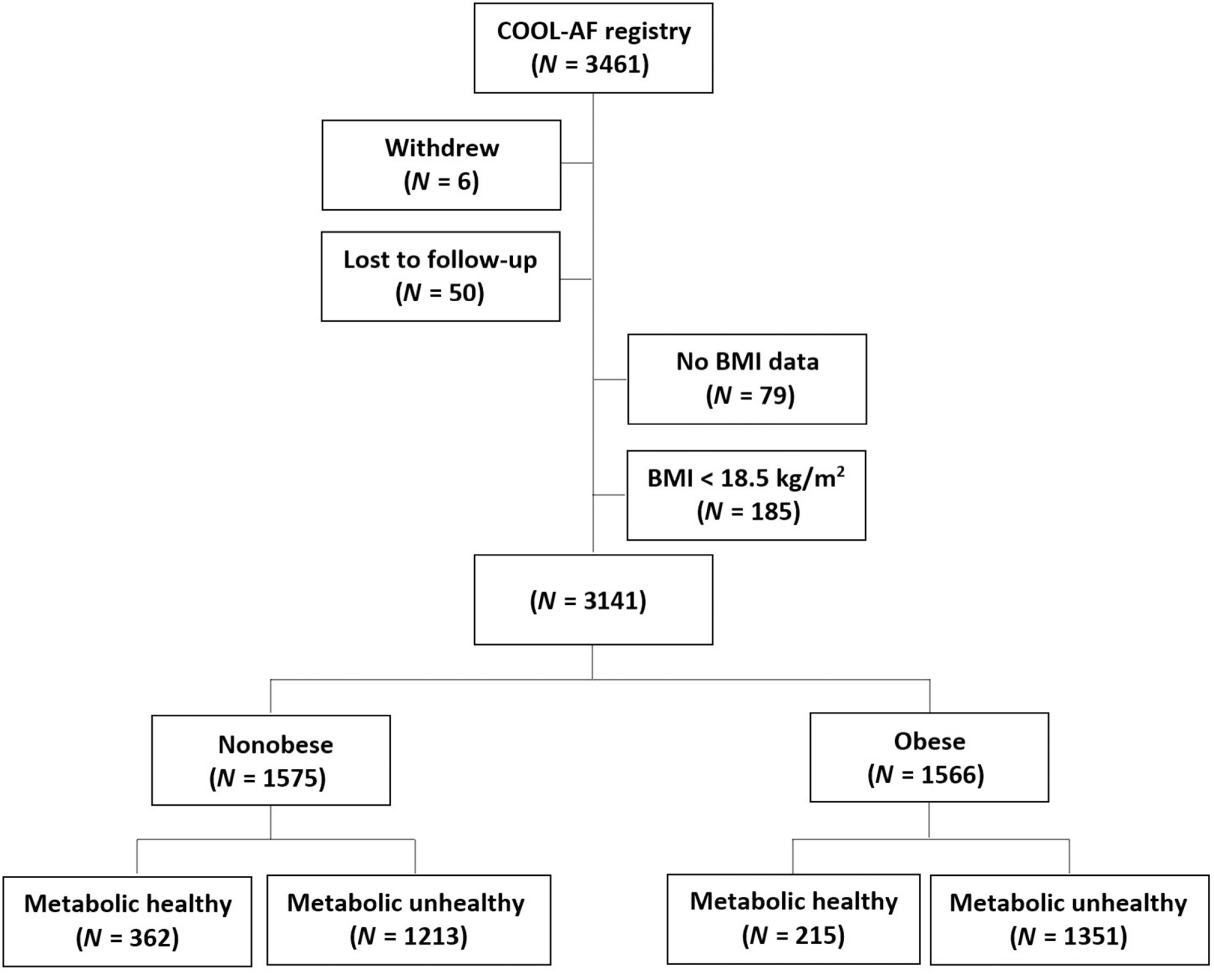
Flow diagram of study population. BMI, body mass index; COOL‐AF, COhort of antithrombotic use and Optimal INR Level in patients with non‐valvular Atrial Fibrillation in Thailand.

Patients were classified into four groups according to their obesity and metabolic status as follows: (a) nonobese and metabolic healthy (*n* = 362, 11.5%), (b) nonobese and metabolic unhealthy (*n* = 1213, 38.6%), (c) obese and metabolic healthy (*n* = 215, 6.9%), and (d) obese and metabolic unhealthy (*n* = 1351, 43.0%). Flow diagram of the study population is shown in Figure 1, with baseline characteristics summarized in Table 1. Patients with metabolic unhealthy were older, more female, more history of coronary artery disease, and higher CHA_2_DS_2_‐VASc score compared to those with metabolic healthy. Patients with obese with metabolic healthy are the youngest group.

**TABLE 1:** Baseline characteristics of study population.

Variables	All (*n* = 3141)	Nonobese and metabolically healthy (*n* = 362)	Nonobese and metabolically unhealthy (*n* = 1213)	Obese and metabolically healthy (*n* = 215)	Obese and metabolically unhealthy (*n* = 1351)	*p* value
Age (years)	67.4 ± 11.1	62.8 ± 12.4	70.8 ± 10.2	59.9 ± 12.2	66.7 ± 10.2	**<.001** ^a^, ^b^, ^c^, ^d^, ^e^, ^f^
Female gender	1289 (41.0%)	123 (34.0%)	510 (42.0%)	82 (38.1%)	574 (42.5%)	**.020** ^a^, ^c^
BMI ≥25	1566 (49.9%)	0 (0.0%)	0 (0.0%)	215 (100.0%)	1351 (100.0%)	**<.001** ^b^, ^c^, ^d^, ^e^
Time after diagnosis of AF (years)	3.4 ± 4.3	3.0 ± 3.8	3.4 ± 4.4	3.2 ± 4.4	3.4 ± 4.4	.356
Atrial fibrillation						.098
Paroxysmal	1084 (34.5%)	146 (40.3%)	415 (34.2%)	66 (30.7%)	457 (33.8%)	
Persistent	602 (19.2%)	56 (15.5%)	223 (18.4%)	49 (22.8%)	274 (20.3%)	
Permanent	1455 (46.3%)	160 (44.2%)	575 (47.4%)	100 (46.5%)	620 (45.9%)	
Symptomatic AF	2417 (77.0%)	294 (81.2%)	928 (76.5%)	175 (81.4%)	1020 (75.5%)	**.049** ^c^
History of heart failure	839 (26.7%)	91 (25.1%)	300 (24.7%)	60 (27.9%)	388 (28.7%)	.120
History of coronary artery disease	517 (16.5%)	36 (9.9%)	234 (19.3%)	14 (6.5%)	233 (17.2%)	**<.001** ^a^, ^c^, ^d^, ^e^, ^f^
Cardiac implantable electronic device	319 (10.2%)	34 (9.4%)	151 (12.4%)	17 (7.9%)	117 (8.7%)	**.008** ^e^
Hypertension	2185 (69.6%)	0 (0.0%)	1012 (83.4%)	0 (0.0%)	1173 (86.8%)	**<.001** ^e^
History of ischemic stroke/TIA	540 (17.2%)	45 (12.4%)	250 (20.6%)	22 (10.2%)	223 (16.5%)	**<.001** ^a^, ^d^, ^e^
Diabetes mellitus	804 (25.6%)	0 (0.0%)	302 (24.9%)	0 (0.0%)	502 (37.2%)	**<.001** ^e^
Smoking	640 (20.4%)	78 (21.5%)	233 (19.2%)	48 (22.3%)	281 (20.8%)	.577
Dyslipidemia	1800 (57.3%)	0 (0.0%)	799 (65.9%)	0 (0.0%)	1001 (74.1%)	**<.001** ^e^
Renal replacement therapy	38 (1.2%)	2 (0.6%)	24 (2.0%)	0 (0.0%)	12 (0.9%)	**.011** ^d^, ^e^
Dementia	28 (0.9%)	0 (0.0%)	18 (1.5%)	0 (0.0%)	10 (0.7%)	**.016** ^a^
History of bleeding	299 (9.5%)	18 (5.0%)	141 (11.6%)	11 (5.1%)	129 (9.5%)	**<.001** ^a^, ^c^, ^f^
CHA_2_DS_2_‐VASc score						**<.001**
0	465 (14.8%)	198 (54.7%)	75 (6.2%)	131 (60.9%)	61 (4.5%)	
1	920 (29.3%)	105 (29.0%)	335 (27.6%)	59 (27.4%)	421 (31.2%)	
≥2	1756 (55.9%)	59 (16.3%)	803 (66.2%)	25 (11.6%)	869 (64.3%)	
HAS‐BLED score						**<.001**
0	467 (14.9%)	101 (27.9%)	98 (8.1%)	57 (26.5%)	211 (15.6%)	
1–2	2191 (69.8%)	234 (64.6%)	866 (71.4%)	147 (68.4)	944 (69.9%)	
≥3	483 (15.4%)	27 (7.5%)	249 (20.5%)	11 (5.1%)	196 (14.5%)	
Antiplatelet	835 (26.6%)	75 (20.7%)	345 (28.4%)	55 (25.6%)	360 (26.6%)	**.034** ^a^
Anticoagulant	2366 (75.3%)	229 (63.3%)	953 (78.6%)	128 (59.5%)	1056 (78.2%)	**<.001** ^a^, ^c^, ^f^
Warfarin	2145 (68.3%)	200 (55.2%)	862 (71.1%)	115 (53.5%)	968 (71.7%)	**<.001** ^a^, ^c^, ^f^
NOACs	221 (7.0%)	29 (8.0%)	91 (7.5%)	13 (6.0%)	88 (6.5%)	.612

*Note*: Bold values indicate statistically significant at *p* < 0.05.

Abbreviations: AF, atrial fibrillation; BMI, body mass index; TIA, transient ischemic attack.

^a^ Nonobese and metabolically healthy vs nonobese and metabolically unhealthy.

^b^ Nonobese and metabolically healthy vs obese and metabolically healthy.

^c^ Nonobese and metabolically healthy vs obese and metabolically unhealthy.

^d^ Nonobese and metabolically unhealthy vs obese and metabolically healthy.

^e^ Nonobese and metabolically unhealthy vs obese and metabolically unhealthy.

^f^ Obese and metabolically healthy vs obese and metabolically unhealthy.

### 3.1 Outcomes

During a mean follow‐up of 32.2 ± 8.3 months, the incidence rate of the composite outcome, all‐cause death, SSE, MI, and HF were 7.21 (6.63–7.82), 3.86 (3.45–4.30), 1.48 (1.23–1.77), 0.47 (0.33–0.64), and 2.84 (2.48–3.23) per 100 person‐years, respectively. The incidence rates of composite outcomes, death, SSE, MI, and HF among the four study groups are shown in Table S1. The overall incidence rates of the primary composite outcome for the nonobese was greater than those obese (8.07 vs 6.37 per 100 person‐years, *p* = .002) and for metabolic unhealthy this was greater than metabolic healthy (7.83 vs 4.57 per 100 person‐years, *p* < .001) (Figure S1).

**FIGURE 2:**
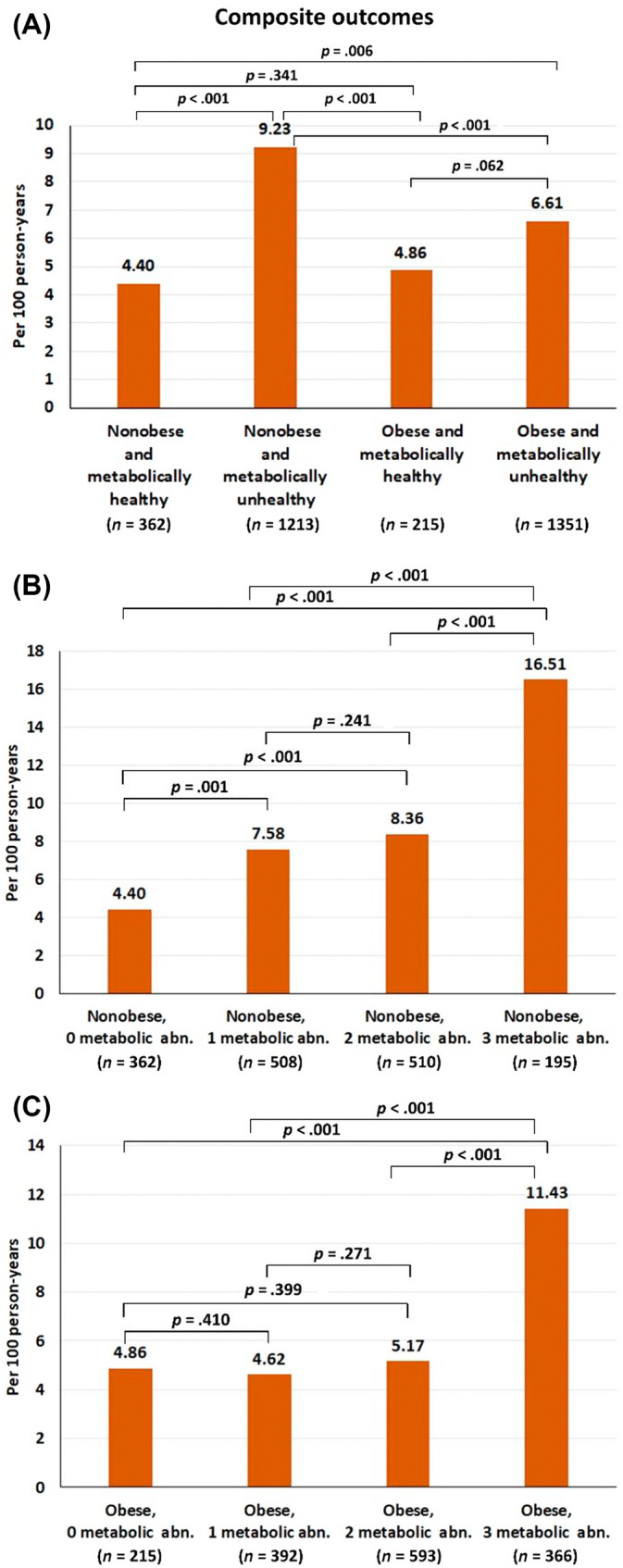
Bar graph of incidence rate of composite outcomes according to (A) four groups of obesity and metabolic status, (B) nonobese with varying degree of metabolic unhealthy, and (C) obese with varying degree of metabolic unhealthy. Abn, abnormalities.

The incidence rate of the composite outcome was highest in the nonobese and metabolic unhealthy group, whereas the metabolic healthy population (obese and nonobese) had a low incidence (Figure 2A). For the nonobese patients, the incidence rate of the composite outcome was significantly increased even with having one metabolic abnormality and markedly increased when they had three metabolic abnormalities (Figure 2B). For the obese patients, the incidence rate of the composite outcome was not significantly increased when they had one or two metabolic abnormalities and only significantly increased with three metabolic abnormalities (Figure 2C). The *p* values for trend for the relation of number of metabolic abnormalities and composite outcomes were <.001 for both nonobese and obese groups. Figure S2 shows cumulative event rate of the four study groups, as well as of the nonobese and obese groups with one, two, and three metabolic abnormalities. The graphs showed similar trends as indicated by the differences in incidence rates among groups.

### 3.2 Multivariable analysis

Age‐ and sex‐adjusted multivariable Cox proportional hazard model analyses demonstrated that metabolic unhealthy nonobese subjects were at higher risk of the composite outcomes more than metabolic unhealthy obese subjects with hazard ratio (HR) 1.39, 95% confidence interval (CI) 1.17–1.66, *p* < .001. Metabolic unhealthy obese subjects had a numerical increased risk of the composite outcomes compared to those metabolic healthy obese (HR 1.36, 95% CI: 0.91–2.02, *p* = 0.133). Metabolic healthy obese subjects were not associated with increased risk.

**FIGURE 3:**
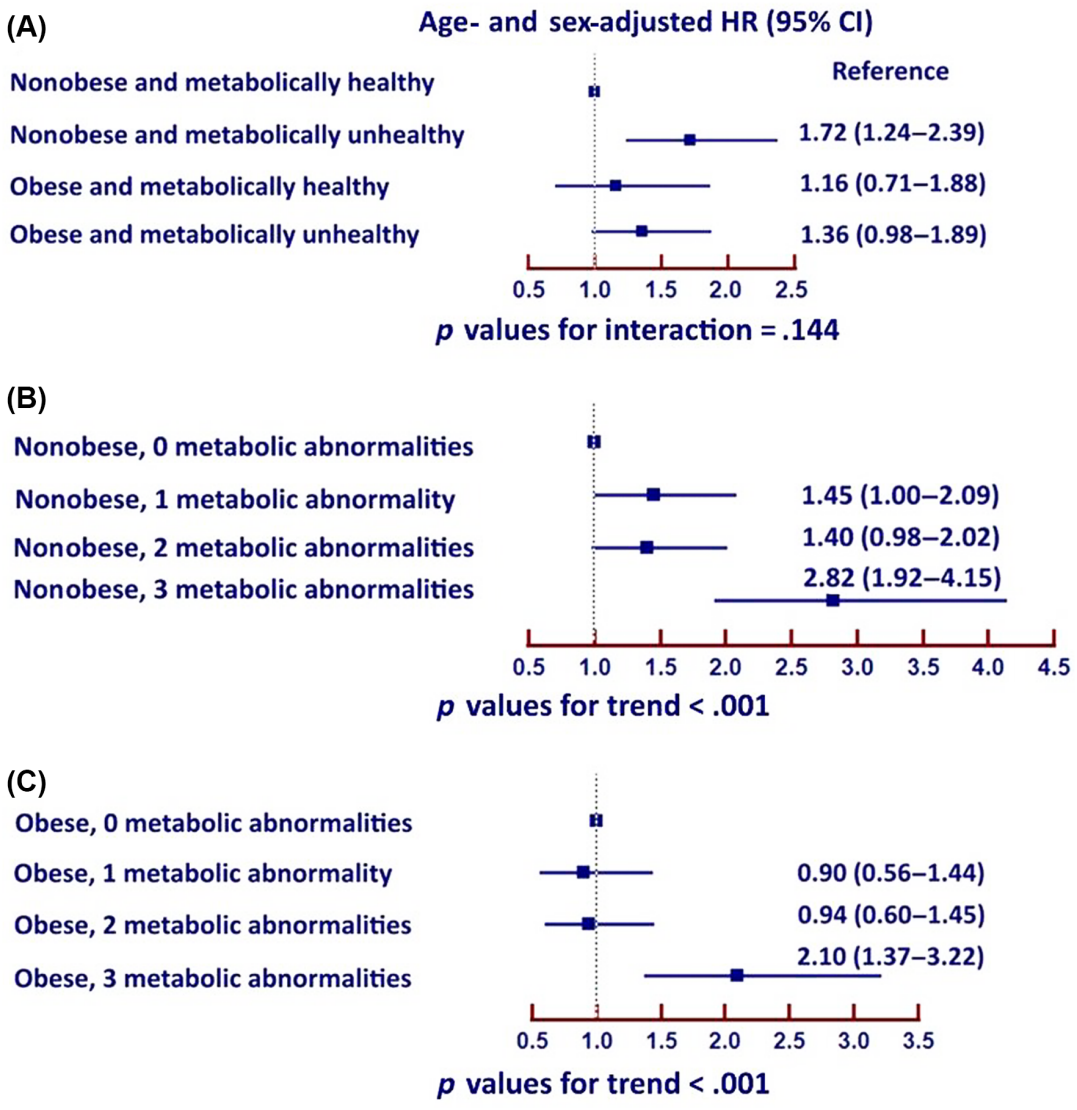
Forest plot of unadjusted age and sex adjusted (left) and adjusted (right) hazard ratio and 95% confidence interval for (A) four groups of obesity and metabolic status, (B) nonobese with varying degree of metabolic unhealthy, and (C) obese with varying degree of metabolic unhealthy. The covariates adjusted in cox models were age ≥ 65 (years), female gender, AF type, symptomatic AF, history of heart failure, history of coronary artery disease, cardiac implantable electrical devices, history of ischemic stroke/transient ischemic attack, smoking, renal replacement therapy, dementia, history of bleeding, antiplatelet, and anticoagulant. AF, atrial fibrillation; CI, confidence interval; HR, hazard ratio.

For nonobese patients, there was a trend that the risk increased when they had one or two metabolic abnormalities and significantly increased when they had three metabolic abnormalities. For obese patients, the risk was similar for patients without metabolic abnormality and those who had one or two metabolic abnormalities but the risk markedly increased when they had three metabolic abnormalities (Figure 3).

### 3.3 Sensitivity analysis

**FIGURE 4:**
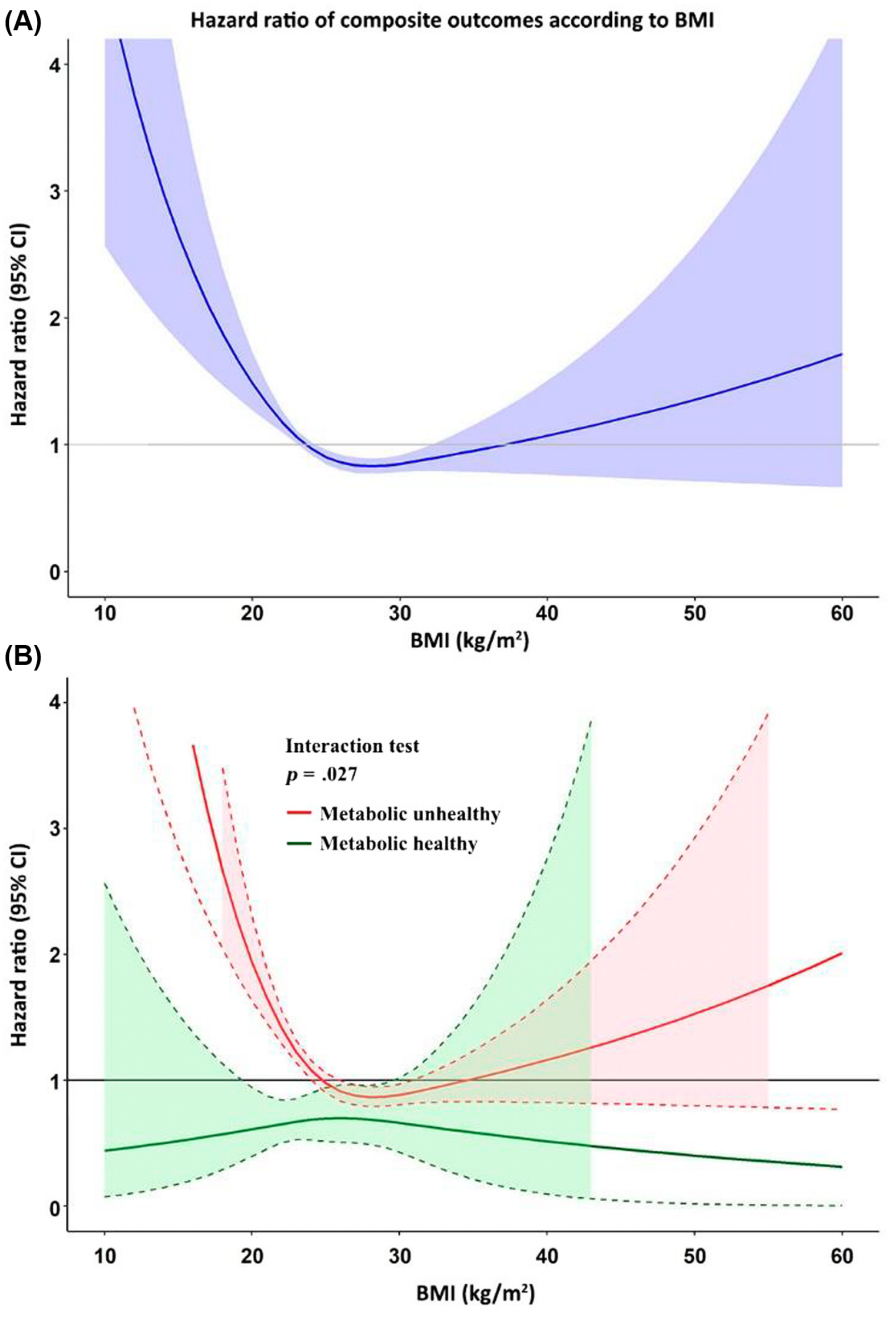
Cubic spline graph showing hazard ratio and 95% confidence interval (CI) of body mass index (BMI) as continuous data with composite outcomes (A) All patients (B) Interaction between patients with metabolic healthy and unhealthy.

Restricted cubic spline graph of BMI on *x*‐axis and hazard ratio of composite outcomes on the *y*‐axis indicated that the risk of composite outcomes was lowest defined as the range of graph where the HR and 95% CI <1.0 in patients with a BMI 25 to 32 kg/m^2^ indicated that (mild) obesity is not adverse (Figure 4A). Nonobese patients had a higher risk than obese patients. An interaction test was performed and shown as the cubic spline of patients with metabolic healthy and unhealthy, where the interaction *p* value was .017 indicating that effect of BMI on composite outcome behaves differently in AF patients who were metabolic healthy and unhealthy.

Figure 4B demonstrates that in AF patients who are metabolically healthy, BMI had no statistically significant effect on clinical outcomes. In those who were metabolic unhealthy, BMI had significant effect on clinical outcomes, whereby BMI <26 kg/m^2^ or >31 kg/m^2^ was associated with an increased risk of clinical outcomes.

We also performed cubic spline graph with the exclusion of extreme case (top and bottom 1%), which is shown in Figure S3. The graph demonstrates a similar finding with the original cubic spline graph.

Stratified analyses by sex (male and female) and age (≥65 and <65 years) was performed. The results of the analyses are shown in Figure S4 and S5. For analyses by sex, the increasing number of metabolic abnormalities increased risk of clinical outcomes both in male and female. However, the difference in the incidence rate of clinical outcomes was more prominent in female than in male. For analyses by age, the incidence rate of clinical outcome was greater in older adults. The increased number of metabolic abnormalities increased risk bot in younger and older adult. The effect of metabolic unhealthy compared to metabolic healthy was greater in older adult compared to younger adult.

## 4 DISCUSSION

The results of this prospective multicenter nationwide study showed that among patients with nonvalvular AF, metabolic unhealthy obese subjects were associated with an increased risk of adverse outcomes in AF patients, whereas metabolically healthy obesity was not associated with an increased risk.

### 4.1 Prevalence of metabolic risk factors in obese and nonobese

The results of our study showed that nearly half of AF patients were obese, with a high proportion of metabolic abnormalities in both obese and nonobese patients. One previous study demonstrated that among French patients who were discharged from hospitals, obesity accounted for 9.5%, and proportions of metabolic healthy was 72.7% in the nonobese group and 32.8% of the obese group.^13^ In our study, obesity accounted for 49.8% of AF patients and metabolic healthy was 23.0% of the nonobese and 13.7% of the obese group. Hence, the majority of our patients were metabolic unhealthy. Another study from general population in Scotland and England showed that metabolic healthy obesity accounted for 5.2% of study group and 21.9% of obese subjects.^14^

Obese patients have an increased risk of many cardiovascular diseases including AF.^3^, ^15^ For example, the Framingham study showed that increased BMI was associated with 5% increased risk of new‐onset AF.^16^ The mechanisms of incident AF in obesity may be related to diastolic dysfunction, increased pericardial fat, inflammatory mediators, structural changes of the atria and concomitant diabetes, obstructive sleep apnea, and concomitant hypertension.^15^ Nonetheless, the cardiovascular risks associated with obesity are not binary (i.e., yes/no) and the “metabolic healthy” obese does not seem to be associated with an increased risk of adverse cardiovascular outcomes.^13^, ^14^ The cardiovascular risks of metabolic healthy obese seem to be similar to metabolic healthy nonobese.

Previous reports showed that obesity increased risk of progression from paroxysmal to permanent AF and increased the risk of AF‐related complications,^3^ whereas data from other studies demonstrated an obesity paradox, whereby obesity was associated with a reduced risk of clinical outcomes compared to the nonobese.^17^ It has been debated whether the obesity paradox is real because many factors have an impact on clinical outcomes and it is unclear which is the causal factor.^18^ The mechanisms underlying the obesity paradox may be the interplay of body fat mass and lean mass, whereby nonsarcopenic obese has a similar risk as healthy individuals whereas sarcopenic obesity would have an increased risk of adverse outcome.^2^

Our study indicates that the optimal BMI for a good clinical outcome in AF patients would be 25–30 kg/m^2^. Other results from the Atrial Fibrillation Follow‐up Investigation of Rhythm Management (AFFIRM) study showed that AF patients with overweight and obesity had a lower all‐cause mortality and cardiovascular mortality compared to non‐obese patients.^17^, ^19^ In the Apixaban for Reduction in Stroke and Other Thromboembolic Events in Atrial Fibrillation (ARISTOTLE) study, AF patients with obesity had a lower risk of ischemic stroke, major bleeding, and all‐cause mortality compared to the nonobese group, especially in women.^19^ Of note, the obesity paradox has also been observed in other cardiovascular disease such as HF.^20^, ^21^ Although obesity is associated with increased risk of HF, the outcomes related to HF may not be related to obesity status.^2^ Indeed, our study shows that metabolic healthy obesity had a similar risk as the metabolic healthy nonobese. The metabolic unhealthy nonobese seems to be the highest risk group.

We indicated in the results and in Supplementary S1 that the overall incidence rates of the primary composite outcome for the nonobese was greater than those obese (8.07 vs 6.37 per 100 person‐years, *p* = .002). From the cubic spline graph (Figure 4A), it may be seen that the HR and 95% CI are <1.0 in patients with a BMI 25 to 32 kg/m^2^. Therefore, the interpretation of this result is that when the BMI is very high or very low, the risk of adverse outcome is increased. The risk is lowest when the BMI is 25–32 kg/m^2^. When we split the cubic spline graph into two groups, metabolic unhealthy (81.6%) and metabolic healthy (18.4%), the finding from Figure 4A is true only for the metabolic unhealthy group. But for the metabolic healthy group, there is no increased risk across the spectrum of BMI. This means that if AF patients keep themselves metabolic healthy, the increase or decrease in BMI had no influence on the risk. Despite the fact that obese AF patients had a lower risk compared to nonobese, the risk for clinical outcomes significantly increased when the BMI was >32 kg/m^2^ as shown in Figure 4A.

### 4.2 Limitations

There are some limitations of this study. First, despite the nationwide multicenter registry, most of the study sites were large or tertiary care hospitals, which may limit generalizability. Second, there might be some selection bias during study enrollment despite instructional that consecutive patients should be enrolled. Third, the definition of obesity in this study was based on BMI only. This is the standard definition suggested by the World Health Organization. Despite the fact that visceral fat or abdominal obesity is an important risk marker, we did not have data on the visceral fat.

## 5 CONCLUSION

Metabolic unhealthy obese subjects were associated with an increased risk of adverse outcomes in AF patients, whereas metabolically healthy obesity was not associated with an increased risk.

## AUTHOR CONTRIBUTIONS

Design: Rungroj Krittayaphong, Arjbordin Winijkul, Conduct/data collection: Rungroj Krittayaphong, Thanita Boonyapiphat, Arjbordin Winijkul, Analysis: Rungroj Krittayaphong, Writing manuscript: Rungroj Krittayaphong, Gregory Y. H. Lip. All authors gave final approval of the version to be published; and agree to be accountable for all aspects of the work.

## FUNDING INFORMATION

This study was funded by a grant from the Health System Research Institute (grant number 59‐053), and the Heart Association of Thailand under the Royal Patronage of H.M. the King. The funder had no role in study design, data collection and analysis, decision to publish, or preparation of the manuscript.

## CONFLICT OF INTEREST STATEMENT

Gregory Y. H. Lip: Consultant and speaker for BMS/Pfizer, Boehringer Ingelheim, Anthos and Daiichi‐Sankyo. No fees are directly received personally. Gregory Y. H. Lip is co‐principal investigator of the AFFIRMO project on multimorbidity in AF, which has received funding from the European Union's Horizon 2020 research and innovation program under grant agreement No 899871. Other authors hereby declare no personal or professional conflicts of interest relating to any aspect of this particular study.

## Supporting information


**Figure S1.** Bar graph of incidence rate of composite outcomes according to obesity (A) and metabolic status (B).
**Figure S2.** Cumulative event rates of the composite outcome.
**Figure S3.** Cubic spline graph showing hazard ratio and 95% confidence interval (CI) of body mass index (BMI) as continuous data with composite outcomes with the exclusion of extreme case (top and bottom 1%) (A) All patients (B) Interaction between patients with metabolic healthy and unhealthy.
**Figure S4.** Bar graph of incidence rate of composite outcomes of four groups of obesity and metabolic status (upper panel), nonobese with varying degree of metabolic unhealthy (middle panel), and obese with varying degree of metabolic unhealthy (lower panel). (A) male (B) female.
**Figure S5.** Bar graph of incidence rate of composite outcomes of four groups of obesity and metabolic status (upper panel), nonobese with varying degree of metabolic unhealthy (middle panel), and obese with varying degree of metabolic unhealthy (lower panel). (A) Age ≥65 years (B) Age <65 years.
**Table S1.** Incidence rate of clinical outcomes according to obesity and metabolic health.

## Data Availability

The dataset that was used to support the results and conclusion of this study are included within the manuscript. The additional data are available from corresponding author upon reasonable request.
