# Supplementary material for: Clinical outcomes of obese and nonobese patients with atrial fibrillation according to associated metabolic abnormalities: A report from the COOL‐AF registry
Source: J Diabetes. 2023 Dec 14;16(7):e13519. doi: 10.1111/1753-0407.13519 (PMC11212287; doi:10.1111/1753-0407.13519)

**Supplementary data**

**Supplementary Table 1.** Incidence rate of clinical outcomes according to obesity and metabolic health

|  | **Number of**  **patients** | **Number of**  **events** | **100 person-**  **years** | **Rate per 100**  **person-years (95% CI)** |
| --- | --- | --- | --- | --- |
| Composite outcomes | 3141 | 575 | 79.75 | 7.21 (6.63-7.82) |
| Non-obese and metabolically healthy | 362 | 42 | 9.54 | 4.40 (3.17-5.95) |
| Non-obese and metabolically unhealthy | 1213 | 276 | 29.89 | 9.23 (8.18-10.39) |
| Obese and metabolically healthy | 215 | 27 | 5.55 | 4.86 (3.21-7.08) |
| Obese and metabolically unhealthy | 1351 | 230 | 34.78 | 6.61 (5.79-7.53) |
| Death | 3141 | 325 | 84.16 | 3.86 (3.45-4.30) |
| Non-obese and metabolically healthy | 362 | 32 | 9.65 | 3.31 (2.27-4.68) |
| Non-obese and metabolically unhealthy | 1213 | 174 | 31.71 | 5.49 (4.70-6.37) |
| Obese and metabolically healthy | 215 | 13 | 5.74 | 2.27 (1.21-3.87) |
| Obese and metabolically unhealthy | 1351 | 106 | 37.07 | 2.86 (2.34-3.46) |
| SSE | 3141 | 123 | 82.97 | 1.48 (1.23-1.77) |
| Non-obese and metabolically healthy | 362 | 6 | 9.59 | 0.63 (0.23-1.36) |
| Non-obese and metabolically unhealthy | 1213 | 64 | 31.09 | 2.06 (1.59-2.63) |
| Obese and metabolically healthy | 215 | 5 | 5.69 | 0.88 (0.29-2.06) |
| Obese and metabolically unhealthy | 1351 | 48 | 36.60 | 1.31 (0.97-1.74) |
| MI | 3141 | 39 | 83.84 | 0.47 (0.33-0.64) |
| Non-obese and metabolically healthy | 362 | 1 | 9.65 | 0.10 (0.003-0.58) |
| Non-obese and metabolically unhealthy | 1213 | 20 | 31.53 | 0.63 (0.39-0.98) |
| Obese and metabolically healthy | 215 | 2 | 5.73 | 0.35 (0.04-1.26) |
| Obese and metabolically unhealthy | 1351 | 16 | 36.92 | 0.43 (0.25-0.71) |
| HF | 3141 | 230 | 81.08 | 2.84 (2.48-3.23) |
| Non-obese and metabolically healthy | 362 | 10 | 9.61 | 1.04 (0.50-1.91) |
| Non-obese and metabolically unhealthy | 1213 | 91 | 30.56 | 2.98 (2.40-3.66) |
| Obese and metabolically healthy | 215 | 11 | 5.61 | 1.96 (0.98-3.51) |
| Obese and metabolically unhealthy | 1351 | 118 | 35.31 | 3.34 (2.77-4.00) |

*Composite outcomes = Death or SSE or MI or HF

SSE = ischemic stroke/systemic embolism, MI = myocardial infarction, HF = heart failure

**Supplementary Figure 1.**

Bar graph of incidence rate of composite outcomes according to obesity (A) and metabolic status (B)


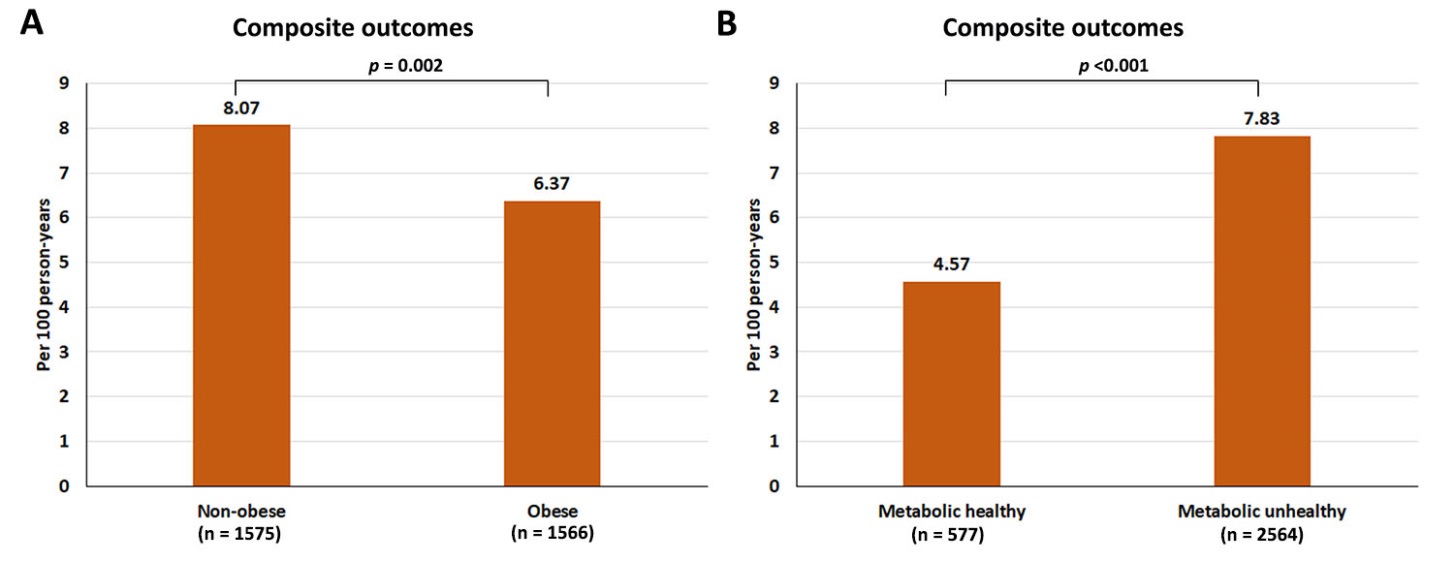


**Supplementary Figure 2.** Cumulative event rates of the composite outcome

A) 4 groups of obesity and metabolic status

B) non-obese with varying degree of metabolic unhealthy

C) obese with varying degree of metabolic unhealthy


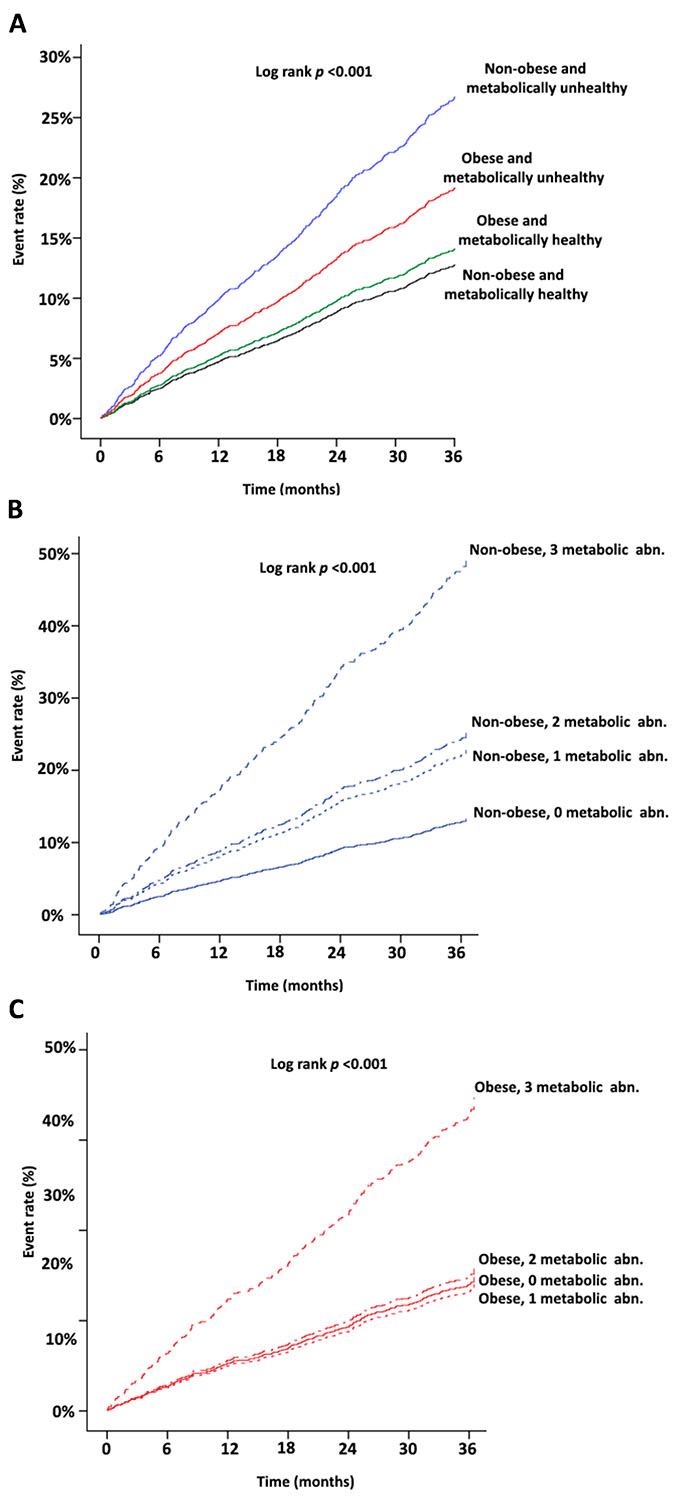


**Supplementary Figure 3.** Cubic spline graph showing hazard ratio and 95% confidence interval (CI) of body mass index (BMI) as continuous data with composite outcomes with the exclusion of extreme case (top and bottom 1%) A. All patients B. Interaction between patients with metabolic healthy and unhealthy.


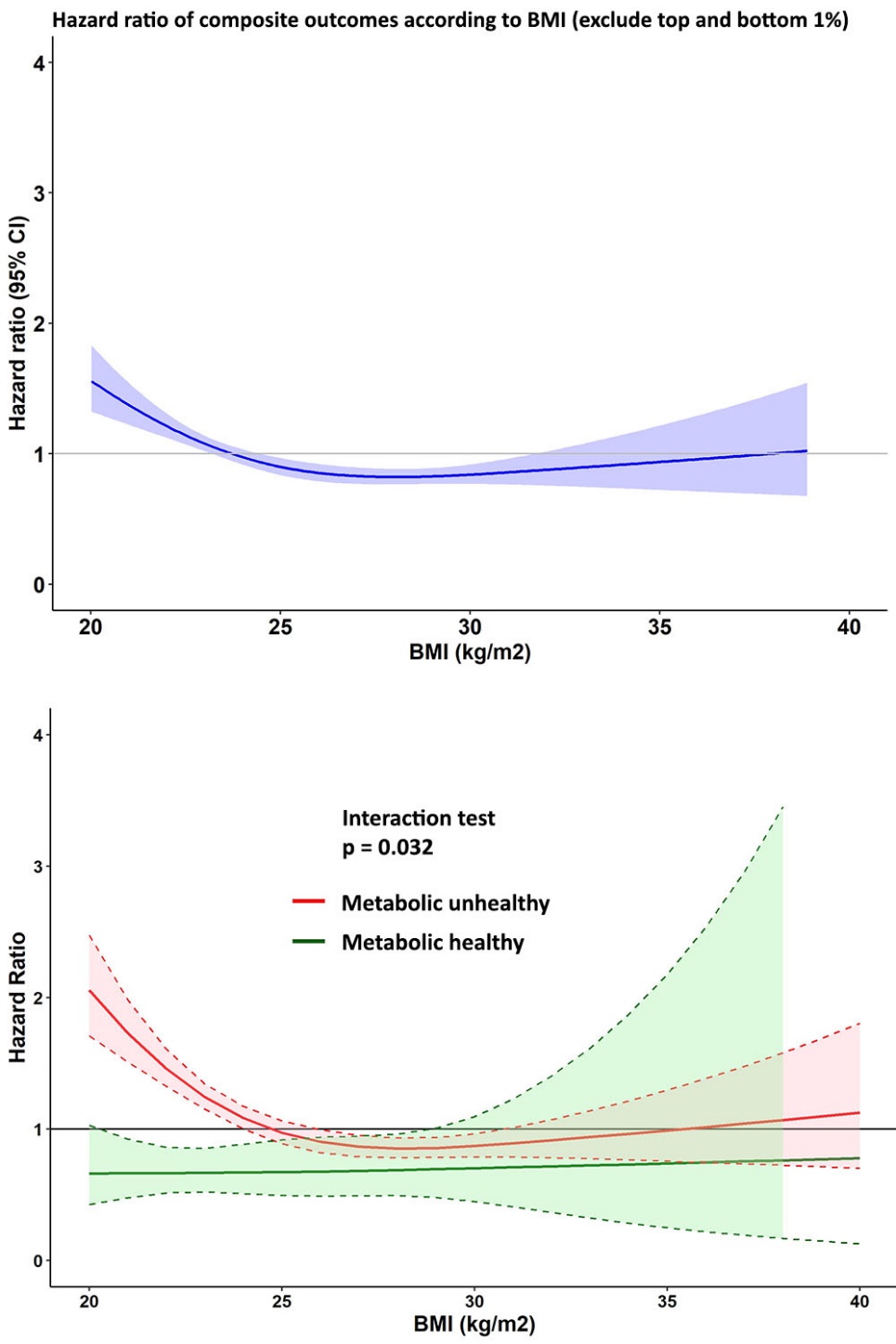


**Supplementary Figure 4.** Bar graph of incidence rate of composite outcomes of 4 groups of obesity and metabolic status (upper panel), non-obese with varying degree of metabolic unhealthy (middle panel), and obese with varying degree of metabolic unhealthy (lower panel). A. male B. female


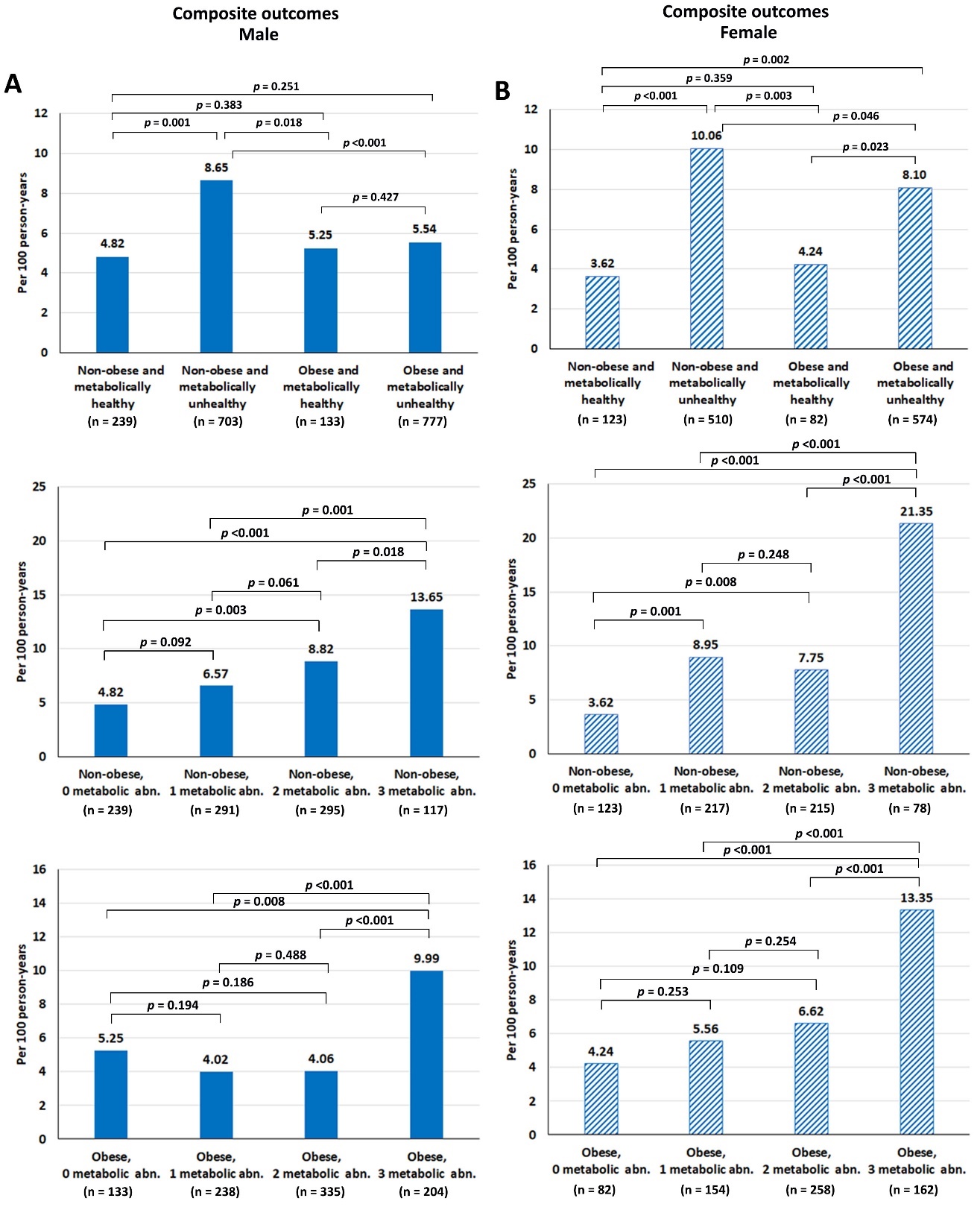


**Supplementary Figure 5.** Bar graph of incidence rate of composite outcomes of 4 groups of obesity and metabolic status (upper panel), non-obese with varying degree of metabolic unhealthy (middle panel), and obese with varying degree of metabolic unhealthy (lower panel). A. Age ≥65 years B. Age < 65 years


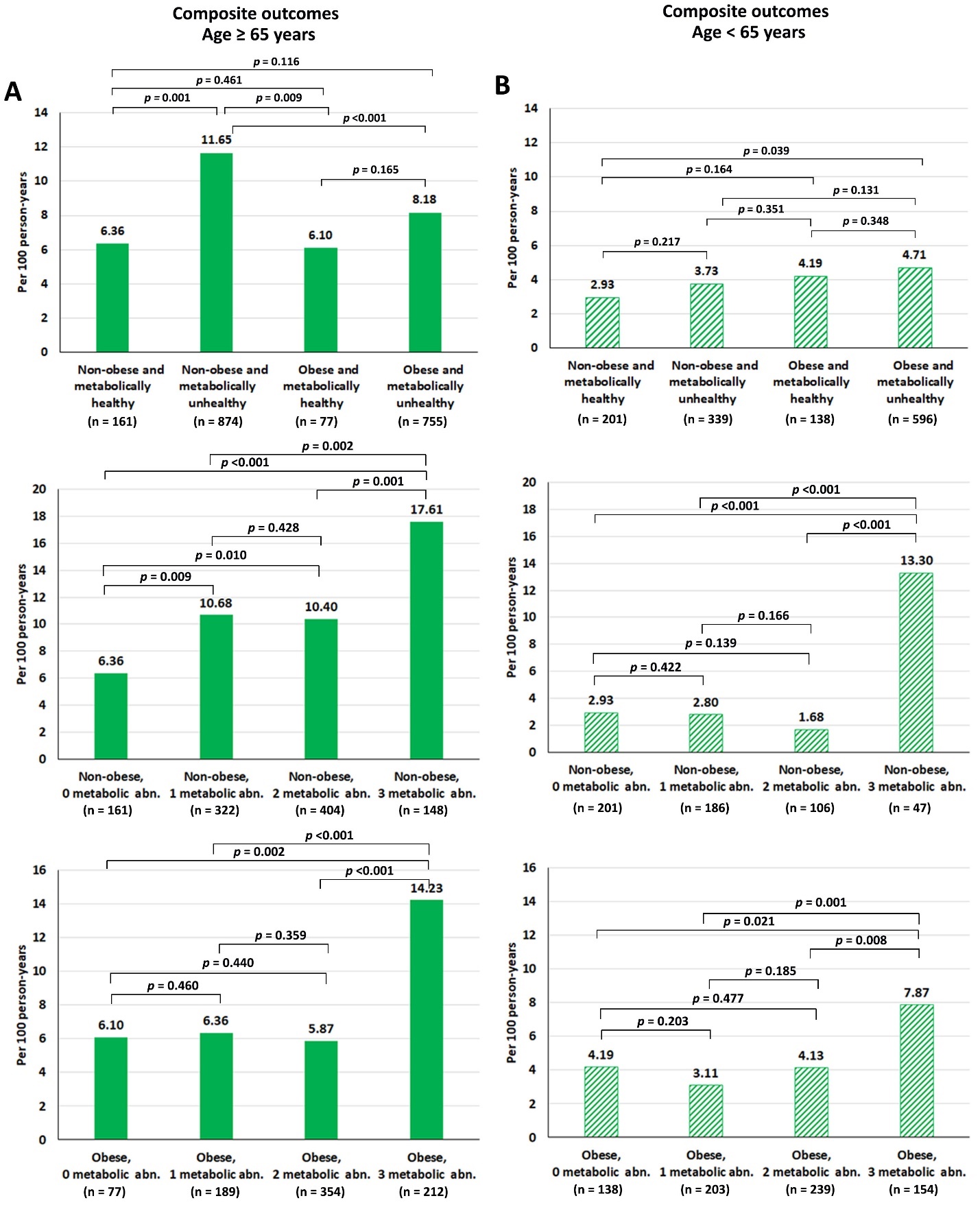

Supplement: Supplementary file 1 — Figure S1. Bar graph of incidence rate of composite outcomes according to obesity (A) and metabolic status (B). Figure S2. Cumulative event rates of the composite outcome. Figure S3. Cubic spline graph showing hazard ratio and 95% confidence interval (CI) of body mass index (BMI) as continuous data with composite outcomes with the exclusion of extreme case (top and bottom 1%) (A) All patients (B) Interaction between patients with metabolic healthy and unhealthy. Figure S4. Bar graph of incidence rate of composite outcomes of four groups of obesity and metabolic status (upper panel), nonobese with varying degree of metabolic unhealthy (middle panel), and obese with varying degree of metabolic unhealthy (lower panel). (A) male (B) female. Figure S5. Bar graph of incidence rate of composite outcomes of four groups of obesity and metabolic status (upper panel), nonobese with varying degree of metabolic unhealthy (middle panel), and obese with varying degree of metabolic unhealthy (lower panel). (A) Age ≥65 years (B) Age <65 years. Table S1. Incidence rate of clinical outcomes according to obesity and metabolic health. [file JDB-16-e13519-s001.docx]
